# Effects of various prosthetic methods for patients with Kennedy Class I partial edentulism on oral hypofunction, subjective symptoms, and oral health-related quality of life

**DOI:** 10.1186/s40729-024-00555-w

**Published:** 2024-06-27

**Authors:** Daisaku Morinaga, Shoji Nagai, Toshio Kaku, Takatoshi Itoh, Yoshiki Soejima, Fumitaka Takeshita, Tadashi Horikawa, Naruyoshi Abe, Toshikazu Iijima, Daigo Soejima, Toshihiro Hara, Ryuta Sato, Mamoru Murakami, Takashi Sawase, Masahiro Nishimura

**Affiliations:** 1 Kyushu Implant Research Group, 4-14 Kokaihonmachi, Chuo-ku, Kumamoto, 860-0851 Japan; 2 Removable Prosthodontics and Implant Dentistry, Advanced Dentistry Center, Kagoshima University Hospital, 8-35-1 Sakuragaoka, Kagoshima, 890-8544 Japan; 3 Nagasaki University Hospital Dental Implant Center, 1-7-1 Sakamoto, Nagasaki, 852-8588 Japan; 4 Department of Fixed Prosthodontics and Orofacial Function, Osaka University Graduate School of Dentistry, 1-8 Yamada-Oka, Suita, 565-0871 Japan

**Keywords:** Oral hypofunction, Oral function, Oral frailty, Implant-supported fixed prosthesis, Removable partial denture

## Abstract

**Purpose:**

This propensity score matching, multicenter, cross-sectional study was performed to examine the effects of various prosthetic methods for dental clinic outpatients with Kennedy Class I partial edentulism (KCIPE) on oral hypofunction, subjective frailty symptoms, and oral health-related quality of life (QOL).

**Methods:**

Patients (n = 348) were classified into the following three groups for analysis: NT, patients with natural dentition providing intermaxillary contact in four occlusal supporting zones; RPD, patients with KCIPE who received removable partial dentures; and ISFP, patients with KCIPE who received implant-supported fixed prostheses. Participants' basic characteristics were recorded, and oral function tests were conducted. Subjective symptoms of physical and oral frailty were investigated via questionnaire. Oral health-related QOL was assessed using the Japanese short version of the Oral Health Impact Profile (OHIP-JP16). Propensity score matching was performed to adjust for patient background factors that could influence oral hypofunction in each group.

**Results:**

Compared with the ISFP group, the RPD group had significantly higher rates of poor oral hygiene, reduced occlusal force, decreased masticatory function, and declines in swallowing function and oral hypofunction; the odds ratio for oral hypofunction was 4.67. Compared with the ISFP group, the RPD group had significantly greater subjective symptoms of physical frailty and oral frailty, as well as higher OHIP scores.

**Conclusions:**

Prosthetic treatment of KCIPE affected oral hypofunction, subjective frailty symptoms, and oral health-related QOL in dental clinic outpatients.

## Background

Dental implant therapy can improve patient quality of life (QOL) by resolving functional and aesthetic disorders related to defects in teeth and periodontal tissue [1, 2]. Selection criteria for dental implant therapy were presented in the McGill consensus statement [1] and the Academy of Osseointegration consensus report [2]. However, selection criteria for implant-supported fixed prostheses (ISFPs) and removable partial dentures (RPDs) among patients with Kennedy Class I partial edentulism (KCIPE), who are frequently encountered in clinical practice, have not been fully established [3].

Previous studies have shown that masticatory function is optimal among patients with natural dentition; it begins to decline among ISFP wearers and worsens among complete denture wearers [4]. Clinical studies comparing ISFP wearers with RPD wearers among partially edentulous patients have shown that ISFP wearers have better masticatory function [5], oral health-related QOL [6–9], and prognosis of adjacent teeth to the intended edentulous space [10, 11]. However, these studies were limited to patients with Kennedy Class II partially edentulous arches or patients with unspecified defect status, and there is no clear evidence concerning the treatment effects of ISFP or RPD on patients with KCIPE.

There is evidence that oral function is associated with physical function [12, 13]. Decreased oral function is a risk factor for physical frailty, sarcopenia, cognitive decline [14–21], and social withdrawal in older adults [22]. Therefore, it is important to maintain oral function when possible; it is also important to identify and treat patients with impaired oral function [21, 22]. To achieve these goals, oral hypofunction was defined by the Japanese Society of Gerodontology using seven tests: oral hygiene, oral dryness, occlusal force, tongue–lip motor function, tongue pressure, masticatory function, and swallowing function [20]. The presence of abnormal results in ≥ 3 of these tests supports a diagnosis of oral hypofunction [20].

Thus far, the effects of various prosthetic treatments for KCIPE on oral hypofunction have been unclear. Additionally, oral hypofunction occurs before oral dysfunction; it comprises mastication disorders and dysphagia [20]. Consequently, in this early stage of dysfunction, subjective symptoms and decreased oral health-related QOL can be missed.

The purpose of this propensity score matching, multicenter, cross-sectional study of dental clinic outpatients was to compare oral hypofunction, subjective frailty symptoms, and oral health-related QOL between patients with KCIPE who received ISFPs and patients with KCIPE who received RPDs.

## Methods

### Participants

This multicenter cross-sectional study was conducted at 14 centers in Japan: Kagoshima University, Nagasaki University, and 12 dental clinics affiliated with the Kyushu Implant Research Group. The inclusion criteria were age ≥ 50 years, current maintenance treatment at one of the 14 centers, and willingness to participate. Patients were excluded if they were currently undergoing dental treatment, had been fitted with a prosthesis for < 1 year, were considered difficult to examine because of psychosis, could not walk unassisted despite caregiver support, or had any of the following conditions: maxillofacial defects, peri-implantitis, temporomandibular joint disorder, cerebrovascular or neuromuscular disease, Sjogren’s syndrome, chronic obstructive pulmonary disease, or a history of radiation therapy.

Written consent was obtained from all patients after they had been informed that their information would be used for research purposes. The study was conducted in full compliance with the World Medical Association’s Declaration of Helsinki.

The survey was conducted from 25 March 2020 to 31 August 2022. In total, 637 patients agreed to participate in the study. Of these patients, the analysis included 348 who had been classified into the following three groups: NT (N = 152), patients with natural dentition providing intermaxillary contact in four occlusal supporting zones in the premolar and molar regions (Eichner Group A); RPD (N = 84), patients with KCIPE who received RPDs on either side of the upper or lower jaw and had natural opposing dentition; and ISFP (N = 112), patients with KCIPE who received ISFPs on either side of the upper or lower jaw and had natural opposing dentition. 289 patients were excluded because they did not match the above inclusion pattern of tooth loss and prosthetic methods (Kennedy class II or class III partial edentulism: 79, Upper and lower jaws or one of them edentulous: 62, ISFP on upper and lower jaws: 100, RPD on upper and lower jaws: 44, No prosthesis for the defect: 4).

This study protocol was approved by the Institutional Review Board of Ethics Committee on Epidemiological Studies, Kagoshima University (approval number: 190224eki) and Nagasaki University Hospital Clinical Research Ethics Committee (approval number: 20032314). This study was conducted according to the principles of the Declaration of Helsinki.

### Basic participant characteristics and oral hypofunction assessment

Basic participant characteristics such as sex, age, height, weight, body mass index (BMI), grip strength, medical history, and oral conditions (e.g., number of remaining teeth) were recorded.

The following seven test items and cut-off values were used to diagnose oral hypofunction [20, 21]: oral hygiene (tongue coating index ≥ 50%), oral dryness using an oral moisture checker (Mucus; Life, Saitama, Japan, oral moisture value < 27.0), occlusal force using a pressure indicating film (Dental Prescale II; GC Corp., Tokyo, Japan, < 500 N), tongue–lip motor function using an automatic counter (KenkoKun Handy, Takei Scientific Instruments Co., Ltd., oral diadochokinetic rate < 6.0 for any of the syllables /pa/, /ta/, or /ka/), tongue pressure using a tongue pressure measurement device (JMS tongue pressure measurement device, JMS Co., Ltd., < 30 kPa), masticatory function using a chewing ability testing system (Gluco Sensor GS-II; GC, < 100 mg/dL), and swallowing function using the self-administered swallowing screening questionnaire (EAT-10: Eating Assessment Tool-10, score ≥ 3). Oral hypofunction was defined as the presence of abnormal results in ≥ 3 of these seven oral function tests [20].

Oral function tests were conducted by dentists or dental hygienists who had received sufficient training and routinely performed such tests. Diagnoses of oral hypofunction were made by dentists.

### Survey of subjective symptoms of physical and oral frailty and measurement of oral health-related QOL

Subjective symptoms of physical and oral frailty were assessed using a questionnaire that comprised five items related to physical frailty (weight, fatigue, grip strength, physical activity, and walking speed) and seven items related to oral frailty (chewing function, swallowing function, and minor oral decline) [23]. Each item was rated on the following 4-point scale: 3, yes; 2, sometimes; 1, tend to; and 0, no. The total score ranged from 0 to 36 points; higher values indicated greater frailty [23]. The total score and the scores for items related to physical frailty and oral frailty were analyzed.

Oral health-related QOL was determined using the Japanese short version of the Oral Health Impact Profile (OHIP-JP16) [24]. Patients rated items on six subscales of the OHIP-JP16 (functional limitations, pain, psychological discomfort, physical disability, psychological disability, and handicap) using a 5-point scale: 0, never; 1, almost never; 2, sometimes; 3, fairly often; and 4, very often. The total score ranged from 0 to 64 points; higher values indicated worse oral health-related QOL. The total score and the scores for each subscale were analyzed.

### Statistical analysis

Sex, age, BMI, grip strength, and systemic diseases reportedly are associated with oral hypofunction and sarcopenia [18, 25, 26]. Therefore, propensity score matching was used to adjust for groupwise differences in these patient background factors i.e. sex, age, BMI, grip strength, and systemic disease [27]. Propensity scores were calculated via logistic regression analysis; these patient background factors were regarded as explanatory variables, whereas prosthetic treatments for KCIPE were regarded as objective variables. Logistic regression model accuracy was evaluated using area under the receiver operating characteristic (ROC) curve values. The caliper was regarded as the standard deviation of the propensity score multiplied by 0.2, and 1:1 propensity score matching was performed.

Patient characteristics and oral function test results were analyzed using the χ^2^ test and odds ratios for categorical variables; they were analyzed using the Mann–Whitney U test for quantitative variables. All statistical analyses were performed using SPSS software (version 29; IBM Japan, Tokyo, Japan), and the significance threshold was set to 0.05.

## Results

Table 1 shows the oral function test results and oral hypofunction diagnoses in the NT and RPD groups after propensity score matching. Compared with the NT group, the RPD group had significantly higher rates of reduced occlusal force, decreased masticatory function, and oral hypofunction than the NT group. The odds ratio for oral hypofunction in the RPD group, relative to the NT group, was 2.06.

**Table 1:** Oral hypofunction test results in the NT and RPD groups

	NT (n = 73)	RPD (n = 73)	p value ^a^	odds ratio (NT/RPD)	95% confidence interval	
	Number (%)	Number (%)			Lower limit	Upper limit
Poor oral hygiene (≥ 50%)	19 (26.0)	28 (38.4)	0.11	1.77	0.88	3.58
Oral dryness (< 27)	20 (27.4)	31 (42.5)	0.06	1.96	0.98	3.91
Reduced occlusal force (< 200 N)	10 (13.7)	32 (43.8)	0.00*	4.92	2.18	11.07
Decreased tongue pressure (< 30 kPa)	39 (53.4)	31 (42.5)	0.16	0.64	0.34	1.24
Decreased masticatory function (< 100 mg/dL)	2 (2.7)	16 (21.9)	0.00*	9.97	2.20	45.14
Deterioration of swallowing function (score ≥ 3)	10 (13.7)	10 (13.7)	1.0	1.0	0.39	2.57
Decreased tongue–lip motor function (< 6 times/s)	49 (67.1)	54 (74.0)	0.36	1.39	0.68	2.85
Oral hypofunction	28 (38.4)	41 (56.2)	0.03*	2.06	1.06	3.99

^a^χ^2^ test

^*^p < 0.05

Table 2 shows the subjective symptoms of physical frailty and oral frailty, as well as OHIP scores, in the NT and RPD groups after propensity score matching. Compared with the NT group, the RPD group had significantly greater subjective symptoms of physical frailty and oral frailty, along with worsened OHIP scores.

**Table 2:** Subjective symptoms and OHIP scores in the NT and RPD groups

	NT (n = 73)	RPD (n = 73)	p value^a^
	Median (Q1, Q3)	Median (Q1, Q3)	
Subjective symptom of physical frailty	8.0 (6.0, 10.5)	10.0 (7.0, 12.5)	0.04*
Subjective symptom of oral frailty	10.0 (8.0, 13.0)	11.0 (9.0, 15.0)	0.02*
Total score of subjective symptoms	18.0 (15.0, 22.5)	21.0 (16.0, 26.0)	0.02*
Functional limitation	0 (0, 2.0)	2.0 (1.0, 4.0)	0.00*
Physical pain	1.0 (0, 2.0)	1.0 (0, 3.0)	0.03*
Psychological discomfort	0 (0, 3.0)	2.0 (0.5, 4.0)	0.00*
Physical disability	0 (0, 2.5)	3.0 (1.0, 5.0)	0.00*
Psychological disability	0 (0, 2.0)	2.0 (0, 3.0)	0.02*
Handicap	0 (0, 1.0)	1.0 (0, 4.0)	0.00*
Total score of OHIP	3.0 (0, 12.0)	14.0 (6.0, 23.0)	0.00*

Q1; 25%tile, Q3; 75%tile

^a^Mann–Whitney U test

^*^p < 0.05

Table 3 shows the oral function test results and oral hypofunction diagnoses in the NT and ISFP groups after propensity score matching. There were no significant differences between the ISFP and NT groups in terms of oral function test results or oral hypofunction diagnoses.

**Table 3:** Oral hypofunction test results in the NT and ISFP groups

	NT (n = 100)	ISFP (n = 100)	p value ^a^	odds ratio (NT/ISFP)	95% confidence interval	
	Number (%)	Number (%)			Lower limit	Upper limit
Poor oral hygiene (≥ 50%)	24 (24.0)	29 (29.0)	0.42	1.29	0.69	2.43
Oral dryness (< 27)	24 (24.0)	29 (29.0)	0.42	1.29	0.69	2.43
Reduced occlusal force (< 200 N)	16 (16.0)	14 (14.0)	0.69	0.86	0.39	1.86
Decreased tongue pressure (< 30 kPa)	46 (46.0)	39 (39.0)	0.32	0.75	0.43	1.32
Decreased masticatory function (< 100 mg/dL)	2 (2.0)	5 (5.0)	0.25	2.58	0.49	13.62
Deterioration of swallowing function (score ≥ 3)	8 (8.0)	6 (6.0)	0.58	0.73	0.25	2.20
Decreased tongue–lip motor function (< 6 times/s)	58 (58.0)	59 (59.0)	0.89	1.04	0.59	1.83
Oral hypofunction	30 (30.0)	31 (31.0)	0.89	1.05	0.57	1.91

^a^χ^2^ test

Table 4 shows the subjective symptoms of physical frailty and oral frailty, as well as OHIP scores, in the NT and ISFP groups after propensity score matching. There were no significant differences between the ISFP and NT groups concerning subjective symptoms of physical frailty and oral frailty, or OHIP scores.

**Table 4:** Subjective symptoms and OHIP scores in the NT and ISFP groups

	NT (n = 100)	ISFP (n = 100)	p value ^a^
	Median (Q1, Q3)	Median (Q1, Q3)	
Subjective symptom of physical frailty	8.0 (6.0, 10.0)	7.5 (5.0, 10.0)	0.55
Subjective symptom of oral frailty	9.0 (7.0, 12.0)	8.5 (7.0, 11.0)	0.46
Total score of subjective symptoms	17.0 (14.0, 22.0)	17.0 (13.0, 21.0)	0.57
Functional limitation	0 (0, 2.0)	0 (0, 2.0)	0.38
Physical pain	1.0 (0, 2.0)	1.0 (0, 2.0)	0.93
Psychological discomfort	0 (0, 2.0)	2.0 (0, 2.0)	0.97
Physical disability	0 (0, 2.0)	0 (0, 2.0)	0.96
Psychological disability	0 (0, 2.0)	0 (0, 2.0)	0.53
Handicap	0 (0, 1.8)	0 (0, 2.0)	0.91
Total score of OHIP	2.5 (0, 12.0)	4.0 (0, 12.0)	0.91

Q1; 25%tile, Q3; 75%tile

^a^Mann–Whitney U test

Table 5 shows the oral function test results and oral hypofunction diagnoses in the ISFP and RPD groups after propensity score matching. Compared with the ISFP group, the RPD group had significantly higher rates of poor oral hygiene, reduced occlusal force, decreased masticatory function, and declines in swallowing function and oral hypofunction. The odds ratio for oral hypofunction in the RPD group, relative to the ISFP group, was 4.67.

**Table 5:** Oral hypofunction test results in the ISFP and RPD groups

	ISFP (n = 71)	RPD (n = 71)	p value ^a^	Odds ratio (ISFP/RPD)	95% confidence interval	
	Number (%)	Number (%)			Lower limit	Upper limit
Poor oral hygiene (≥ 50%)	12 (16.9)	24 (33.8)	0.02*	2.51	1.14	5.54
Oral dryness (< 27)	20 (28.2)	30 (42.3)	0.08	1.87	0.93	3.76
Reduced occlusal force (< 200 N)	11 (15.5)	28 (39.4)	0.00*	3.55	1.60	7.90
Decreased tongue pressure (< 30 kPa)	29 (40.8)	30 (42.3)	0.87	1.06	0.54	2.07
Decreased masticatory function (< 100 mg/dL)	3 (4.2)	16 (22.5)	0.00*	6.59	1.83	23.80
Deterioration of swallowing function (score ≥ 3)	2 (2.8)	8 (11.3)	0.04*	4.38	0.90	21.41
Decreased tongue–lip motor function (< 6 times/s)	41 (57.7)	50 (70.4)	0.12	1.74	0.87	3.49
Oral hypofunction	7 (9.9)	24 (33.8)	0.01*	4.67	1.86	11.74

^a^χ^2^ test

^*^p < 0.05

Table 6 shows the subjective symptoms of physical frailty and oral frailty, as well as OHIP scores, in the ISFP and RPD groups after propensity score matching. Compared with the ISFP group, the RPD group had significantly greater subjective symptoms of physical frailty and oral frailty, along with worsened OHIP scores.

**Table 6:** Subjective symptoms and OHIP scores in the ISFP and RPD groups

	ISFP (n = 71)	RPD (n = 71)	p value ^a^
	Median (Q1, Q3)	Median (Q1, Q3)	
Subjective symptom of physical frailty	8.0 (6.0, 9.0)	10.0 (7.0, 12.0)	0.00*
Subjective symptom of oral frailty	8.0 (7.0, 11.0)	110 (9.0, 15.0)	0.00*
Total score of subjective symptoms	16.0 (13.0, 20.0)	21.0 (16.0, 26.0)	0.00*
Functional limitation	0 (0, 2.0)	2.0 (1.0, 4.0)	0.00*
Physical pain	0 (0, 2.0)	1.0 (0, 3.0)	0.01*
Psychological discomfort	0 (0, 2.0)	3.0 (1.0, 5.0)	0.00*
Physical disability	0 (0, 1.0)	3.0 (1.0, 5.0)	0.00*
Psychological disability	0 (0, 2.0)	2.0 (0, 4.0)	0.00*
Handicap	0 (0, 2.0)	1.0 (0, 4.0)	0.00*
Total score of OHIP	3.0 (0, 9.0)	14.0 (6.0, 24.0)	0.00*

Q1; 25%tile, Q3; 75%tile

^a^Mann–Whitney U test

^*^p < 0.05

Appendix Table 7 shows the basic participant characteristics before and after propensity score matching in the NT and RPD groups. Before propensity score matching, there were 152 patients in the NT group and 84 patients in the RPD group. There were significant differences between the two groups in all basic participant characteristics except grip strength. After propensity score matching, there were 73 participants in both the NT and RPD groups; no significant differences between the two groups were observed regarding basic participant characteristics. Stander deviation of propensity score and caliper were 0.15 and 0.03, respectively.

Appendix Table 8 shows the basic participant characteristics before and after propensity score matching in the NT and ISFP groups. Before propensity score matching, there were 152 patients in the NT group and 112 patients in the ISFP group. Significant differences in BMI and grip strength were observed between the two groups. After propensity score matching, there were 100 participants in both the NT and ISFP groups; no significant differences between the two groups were observed regarding basic participant characteristics. Stander deviation of propensity score and caliper were 0.14 and 0.03, respectively.

Appendix Table 9 shows the basic participant characteristics before and after propensity score matching in the ISFP and RPD groups. Before propensity score matching, there were 112 patients in ISFP group and 84 patients in the RPD group. Significant differences in age and number of underlying diseases were observed between the two groups. After propensity score matching, there were 71 participants in both the ISFP and RPD groups; no significant differences between the two groups were observed regarding basic participant characteristics. Stander deviation of propensity score and caliper were 0.15 and 0.03, respectively.

In all analyses, the area under the ROC was 0.7 (95% confidence interval, 0.6–0.7), and the logistic regression analysis used for propensity score matching demonstrated fair accuracy.

## Discussion

This propensity score matching, multicenter, cross-sectional study explored the effects of various prosthetic treatments for KCIPE on oral hypofunction, subjective frailty symptoms, and oral health-related QOL among dental clinic outpatients. Compared with the RPD group, the ISFP group exhibited superior oral function, fewer subjective symptoms, and better oral health-related QOL, revealing how prosthetic methods affect these parameters.

The choice of ISFP or RPD for a patient with KCIPE is based on that patient's oral status, living situation, general condition, economic status, and personal preferences [3]. To our knowledge, this study is the first to provide clear evidence concerning the therapeutic efficacies of ISFP and RPD in patients with KCIPE.

The present results are highly accurate because we specified the defect types and explored the effects of prosthetic methods on those defects. The multicenter design ensured a large sample size and avoided bias. Although the cross-sectional nature of the study precluded random assignment and may have introduced confounding factors, propensity score matching enabled estimation of causal effects by adjusting for biases that could influence the findings.

Sample sizes after propensity score matching vary among studies because of population-related differences in propensity scores. Prior to study completion, we could not predict the sample size after propensity score matching. However, after propensity score matching, we performed sample size analysis using G* Power software (version 3.1.9.4); we assumed that the mean difference in QOL score between ISFP and RPD groups would be 0.82, based on previous findings [8]. The analysis revealed that the minimum sample size was N = 42 [alpha, 0.05; beta, 0.05 (95% power)], indicating that our sample sizes were sufficient.

Factors affecting post-treatment oral function (e.g., masticatory function and occlusal force) include clinical difficulty. In the treatment of partially edentulous patients, Prosthetic treatment difficulty indices developed by the Japan Prosthodontic Society indicate that, among partially edentulous patients, it is most difficult to treat patients with KCIPE who display premolar and molar defects [28]. In the present study, we did not specify the defect size. The participants were patients who had been fitted with a dental prosthesis for ≥ 1 year and had undergone regular maintenance treatment without problems. Therefore, we suspect that pre-treatment clinical difficulty levels were randomized via propensity score matching. However, confounding bias cannot be excluded with respect to factors that were not regarded as covariates when calculating propensity scores. Unadjusted factors may include general health conditions (cognitive function, skeletal muscle mass, and nutritional status) and living situation [18, 19, 22]. Additionally, the present study did not include participants with substantial impairment concerning physical function or oral function, which may limit the generalizability of the findings.

To our knowledge, few clinical studies have compared ISFP wearers and RPD wearers [5–8]. Akagawa et al. used electromyography to examine chewing function during RPD wear and ISFP wear in patients with Kennedy Class II partially edentulous mandibles; they found that ISFP wearers had greater masticatory muscle activity and better masticatory function, compared with RPD wearers [5]. Kapur et al., Kuboki et al., Furuyama et al., and Kurosaki et al. reported that ISFP wearers had better oral health-related QOL than RPD wearers in studies of patient-oriented outcomes [6–9]. Yamazaki et al. observed earlier loss of adjacent teeth to intended edentulous space in RPD wearers than in ISFP wearers [10, 11]. However, these studies were limited to patients with Kennedy Class II partially edentulous arches or patients with unspecified defect status, limiting their generalizability to the selection of optimal prosthetic treatments for KCIPE. In the present study, seven types of oral function were comprehensively evaluated in patients with KCIPE; the results showed that odds ratios for oral hypofunction in the RPD group were 2.06 (compared with the NT group) and 4.67 (compared with the ISFP group). Although the comparison groups were not identical, these results suggest that ISFPs are effective for the prevention of oral decline in patients with KCIPE.

Aspects of oral function, such as the number of remaining teeth and chewing ability, are associated with oral health-related QOL [29–31]. Therefore, oral health maintenance is essential for improving oral health-related QOL. Comprehensive assessments of oral function revealed that RPD group had significantly worse results concerning multiple aspects of oral function compared with the other groups; it also had significantly lower oral health-related QOL. Kodama et al. investigated the relationship between oral hypofunction and oral health-related QOL in community residents aged ≥ 65 years [32]; they found that oral health-related QOL decreased as the number of functional impairments increased, consistent with the present results.

The OHIP-49 was originally developed to measure oral health-related QOL [33], however the OHIP-14 was developed for response time and convenience [34]. The OHIP-14 has seven dimensions, including social disability. In this study, however, we decided to use the OHIP-JP16, which does not include social disability but is specialized for comparing before and after prosthetic treatment [24].

In previous cohort studies involving community residents, the incidences of oral hypofunction ranged from 42.7 to 62.9% [21, 35–37]. Hatanaka et al. reported that the incidence of oral hypofunction was 63.4% among older outpatients in a university hospital [38]. Ozaki et al. reported that the incidence of oral hypofunction was 89.8% among older people requiring nursing care [39]. In the present study, the RPD group had the highest incidence of oral hypofunction (28%); this value is considerably lower than the values in previous reports. These findings suggest that the incidence of oral hypofunction varies according to the characteristics of the study population. The low incidence of oral hypofunction in the present study may be attributed to the patients’ regular management at dental clinics, as well as the exclusion of patients undergoing dental treatment or displaying substantial functional decline.

Oral frailty reportedly involves slight declines in oral function, such as decreased tongue movement, food spillage, and mild choking [20]. Kugiyama et al. reported that oral frailty was a risk factor for physical frailty and death in a longitudinal study of community-dwelling older adults [15]. There is evidence that oral frailty and oral hypofunction have many overlapping aspects and cannot be easily distinguished [20]. To our knowledge, no previous study used questionnaires to assess subjective symptoms of oral hypofunction. In the present study, we used an existing questionnaire [24] to investigate subjective symptoms of physical and oral frailty. This questionnaire was previously used by Hihara et al. who reported that oral frailty tended to increase with age in a population of 1214 individuals [24]. Our analysis involved propensity score matching to adjust for age differences among groups; thus, we could not assess the relationship between age and subjective symptoms of frailty. However, our results indicated that scores concerning subjective symptoms of frailty and oral health-related QOL were similar to the incidence of oral hypofunction. These results highlight the importance of regular management and maintenance of oral function, with attention to subjective symptoms of frailty and oral health-related QOL; such efforts can facilitate early detection of oral hypofunction.

## Conclusions

Compared with the RPD group, the ISFP group exhibited superior oral function, fewer subjective symptoms, and better oral health-related QOL, revealing how prosthetic methods affect these parameters in patients with KCIPE.

## Data Availability

The data sets generated and analyzed during the current study are available from the corresponding author on reasonable request.

## Appendix

See Tables 7, 8, 9.

**Table 7:** Participant characteristics before and after propensity score matching in the NT and RPD groups

	Before propensity score matching			After propensity score matching		
	NT (n = 152)	RPD (n = 84)	p value	NT (n = 73)	RPD (n = 73)	p value
Women/Men: Number (%)	111 (73)/41 (27)	48 (57.1)/36 (42.9)	0.01^a^*	44 (60.3)/29 (39.7)	44 (60.3)/29 (39.7)	1.0^a^
Age: Median (Q1, Q3)	72.0 (64.0, 77.8)	74.0 (68.0, 80.0)	0.02^b^*	74.0 (65.0, 80.5)	73.0 (67.5, 80.0)	0.75^b^
BMI (kg/m^2^): Median (Q1, Q3)	21.9 (20.3, 23.8)	23.3 (21.2, 25.0)	0.00^b^*	22.9 (21.3, 24.7)	22.8 (20.7, 24.8)	0.87^b^
grip strength (kgf): Median (Q1, Q3)	23.7 (19.9, 28.0)	24.1 (18.6, 32.1)	0.81^b^	23.7 (19.7, 32.4)	24.2 (19.1, 32.7)	0.88^b^
Number of underlying diseases: Median (Q1, Q3)	1.0 (0, 1.0)	1.0 (0, 2.0)	0.02^b^*	1.0 (0, 2.0)	1.0 (0, 2.0)	0.36^b^

Q1; 25%tile, Q3; 75%tile

^a^χ^2^ test

^b^Mann–Whitney U test

^*^p < 0.05

**Table 8:** Participant characteristics before and after propensity score matching in the NT and ISFP groups

	B**e**fore propensity score matching			After propensity score matching		
	NT (n = 152)	ISFP (n = 112)	p value	NT (n = 100)	ISFP (n = 100)	p value
Women/Men: Number (%)	111 (73.0)/41 (27.0)	72 (64.3)/40 (35.7)	0.08^a^	66 (66.0)/34 (34.0)	66 (66.0)/34 (34.0)	1.0^a^
Age: Median (Q1, Q3)	72.0 (64.0, 77.8)	71.5 (66.3, 76.8)	0.67^b^	72.0 (64.0, 78.0)	71.0 (66.3, 77.0)	0.77^b^
BMI (kg/m^2^): Median (Q1, Q3)	21.9 (20.3, 23.8)	23.0 (21.7, 25.0)	0.00b*	22.9 (21.6, 24.6)	23.0 (21.6, 24.6)	0.85^b^
grip strength (kgf): Median (Q1, Q3)	23.7 (19.9, 28.0)	26.0 (21.6, 30.2)	0.01^b^*	24.6 (20.8, 34.0)	26.0 (21.3, 30.2)	0.53^b^
Number of underlying diseases: Median (Q1, Q3)	1.0 (0, 1.0)	1.0 (0, 2.0)	0.21b	0 (0, 1.0)	1.0 (0, 1.0)	0.63^b^

Q1; 25%tile, Q3; 75%tile

^a^χ^2^ test

^b^Mann–Whitney U test

^*^p < 0.05

**Table 9:** Participant characteristics before and after propensity score matching in the ISFP and RPD groups

	Before propensity score matching			After propensity score matching		
	ISFP (n = 112)	RPD (n = 84)	p value	ISFP (n = 71)	RPD (n = 71)	p value
Women/Men: Number (%)	72 (62.6)/40 (37.4)	48 (57.1)/36 (42.9)	0.31^a^	43 (60.6)/28 (39.4)	43 (60.6)/28 (39.4)	0.49^a^
Age: Median (Q1, Q3)	71.5 (66.3, 76.8)	74.0 (68.0, 80.0)	0.03^b^*	72.0 (67.0, 77.0)	72.0 (67.0, 79.0)	0.59^b^
BMI (kg/m^2^): Median (Q1, Q3)	23.0 (21.7, 25.0)	23.3 (21.2, 25.0)	0.87^b^	23.4 (21.8, 25.0)	23.0 (21.0, 25.1)	0.42^b^
grip strength (kgf): Median (Q1, Q3)	26.0 (21.6, 30.2)	24.1 (18.6, 32.1)	0.12^b^	25.0 (20.9, 27.7)	24.2 (19.5, 32.6)	0.84^b^
Number of underlying diseases: Median (Q1, Q3)	0 (0, 1.0)	1.0 (0, 2.0)	0.00^b^*	1.0 (0, 2.0)	1 (0, 2.0)	0.85^b^

Q1; 25%tile, Q3; 75%tile

^a^χ^2^ test

^b^Mann–Whitney U test

*p < 0.05

## Abbreviations

NT: Natural teeth
ISFP: Implant-supported fixed prosthesis
RPD: Removable partial denture
KCIPE: Kenney Class I partial edentulism
QOL: Quality of life
BMI: Body mass index
OHIP: Oral Health Impact Profile
EAT-10: Eating Assessment Tool-10
ROC: Receiver operating characteristic
